# Targeting Protein Quality Control Mechanisms by Natural Products to Promote Healthy Ageing

**DOI:** 10.3390/molecules23051219

**Published:** 2018-05-19

**Authors:** Sophia Wedel, Maria Manola, Maria Cavinato, Ioannis P. Trougakos, Pidder Jansen-Dürr

**Affiliations:** 1 Institute for Biomedical Aging Research, University of Innsbruck, 6020 Innsbruck, Austria; Sophia.Wedel@uibk.ac.at (S.W.); Maria.Cavinato-Nascimento@uibk.ac.at (M.C.); 2 Department of Cell Biology and Biophysics, Faculty of Biology, National and Kapodistrian University of Athens, 15784 Athens, Greece; mmanola@biol.uoa.gr

**Keywords:** autophagy, proteasome, proteostasis network, natural products, Nrf2

## Abstract

Organismal ageing is associated with increased chance of morbidity or mortality and it is driven by diverse molecular pathways that are affected by both environmental and genetic factors. The progression of ageing correlates with the gradual accumulation of stressors and damaged biomolecules due to the time-dependent decline of stress resistance and functional capacity, which eventually compromise cellular homeodynamics. As protein machines carry out the majority of cellular functions, proteome quality control is critical for cellular functionality and is carried out through the curating activity of the proteostasis network (PN). Key components of the PN are the two main degradation machineries, namely the ubiquitin-proteasome and autophagy-lysosome pathways along with several stress-responsive pathways, such as that of nuclear factor erythroid 2-related factor 2 (Nrf2), which mobilises cytoprotective genomic responses against oxidative and/or xenobiotic damage. Reportedly, genetic or dietary interventions that activate components of the PN delay ageing in evolutionarily diverse organisms. Natural products (extracts or pure compounds) represent an extraordinary inventory of highly diverse structural scaffolds that offer promising activities towards meeting the challenge of increasing healthspan and/or delaying ageing (e.g., spermidine, quercetin or sulforaphane). Herein, we review those natural compounds that have been found to activate proteostatic and/or anti-stress cellular responses and hence have the potential to delay cellular senescence and/or in vivo ageing.

## 1. Introduction

The viability of metazoans largely depends on their ability to regulate metabolic processes in order to generate biomolecules [1]. In eukaryotic cells these molecules are produced in mitochondria through the action of a protein machinery that drives the oxidative phosphorylation (OXPHOS) [2]. During this process, reactive oxygen species (ROS) are formed as by-products [3]. Free radicals and their derivatives are highly reactive molecules and, in physiological concentrations, are essential for proper intracellular signalling, metabolism and responses to pathogens. If their concentration exceeds the cellular antioxidant capacity, however, they cause oxidative stress and damage to all cellular biomolecules [4]. In addition to mitochondria, ROS are also produced by NAD(P)H oxidases, xanthine oxidase and nitric oxide synthase, and may also arise from exogenous sources such as atmospheric pollutants, ultraviolet (UV) light, X- or gamma-rays [3,4,5].

Organisms maintain a proper supply of energetic molecules and retain low levels of stressors or damaged biomolecules via the action of a complex quality control system [6]. At the proteome level, this process is largely achieved via the action of the highly integrated and modular proteostasis network (PN) [7,8,9,10]. During the ageing process, cellular functions deteriorate and compromise these mechanisms, resulting in the impairment of signalling, repair and clearance pathways [11]. This promotes the gradual accumulation of stressors, which correlates with increased disability, morbidity and inevitably death [11,12]. In accordance with this view, age is the major risk factor for several life-threatening diseases, including cancer, cardiovascular diseases, neurodegeneration and diabetes [13,14]. Interestingly, ageing can be delayed by either dietary [e.g., caloric restriction (CR)] or genetic interventions, which modulate stress-responsive pathways and thus affect, either directly or indirectly, the impact of stressors and the rate that damaged biomolecules accumulate [15].

Herein, we summarise the most recent findings related to natural compounds that reportedly delay cellular senescence and/or prolong in vivo longevity by activating cytoprotective proteostatic mechanisms and/or the nuclear factor E2-related factor (Nrf2) anti-stress pathway.

## 2. Overview of the Cellular Proteostatic Modules

Downstream to genetic information, there is a world of immense complexity and plasticity, namely the proteome. The entry point in this world occurs via ribosome-mediated protein synthesis that takes place in both the cytosol and the endoplasmic reticulum (ER). As the average proteome size has increased during evolution [16] and the consequences of an unstable proteome can be catastrophic [17], cells have evolved a system that ensures proteome stability, namely the PN [6]. The PN machinery comprises numerous chaperones, folding enzymes, trafficking and degradation components [6]. During conditions of proteotoxic stress, the PN determines the fate of damaged polypeptides by either folding, holding, or degrading [18].

The PN is regulated at organismal, tissue-specific and cellular level [10]. The protein synthesis module along with the machineries involved in sorting and trafficking of newly synthesised polypeptides are key components of the PN and they are complemented by the unfolded protein response of the ER (UPR^ER^) and mitochondria (UPR^mt^), the intra- and extra-cellular molecular chaperones and a number of compartmentalised proteases, along with the two main degradation branches, i.e., the ubiquitin-proteasome system (UPS) and the autophagy-lysosome (ALP) pathway [9,10,19,20].

A number of short-lived transcription factors are also considered to be part of the PN as they mobilise genomic cytoprotective responses [21]. These, among many others, include heat shock factor 1 (Hsf1), which regulates the levels of molecular chaperones [22]; forkhead box O (FoxO), which promotes antioxidant and metabolic genomic responses [23], and Nrf2, which responds to oxidative, electrophilic, and/or proteotoxic stress [24].

Deregulation of the PN functionality is associated with ageing and it is considered a major risk factor for a wide spectrum of age-related protein conformational diseases such as immunological and metabolic disorders, cardiovascular and neurodegenerative diseases and cancer [8,25,26]. Moreover, loss of proteostasis is recognised as a hallmark of ageing, indicating the great significance of the PN in cellular functionality and survival [27]. Additionally, several studies have revealed that the activation of proteostatic modules by genetic, dietary (e.g., CR), and/or pharmacological interventions increases organismal health- and/or life-span and delays cellular senescence [15]. For example, UPS activation is able to delay ageing in numerous cellular models [9]. Also, genetic activation of the 20S proteasome leads to lifespan extension and increases stress resistance in the nematodes *Caenorhabditis elegans* [28].

## 3. The Ubiquitin-Proteasome System (UPS)

The UPS degrades short-lived, poly-ubiquitinated normal proteins and non-functional or misfolded polypeptides. Ubiquitinated polypeptides are degraded by the 26S proteasome, while non-native (e.g., oxidised) polypeptides are likely degraded by the 20S proteasome via chaperone-mediated targeting [29]. The ubiquitin-proteasome system is composed of ubiquitin-activating, conjugating and ligating enzymes and the 26S proteasome [9]. The 26S eukaryotic proteasome is a protein machine of ~2.5 MDa that is composed of a 20S core particle (CP) to which one or two 19S cap regulatory particles (RP) are bound [30,31]. The 20S CP consists of four stacked heptameric rings (two α-type surrounding two β-type rings) that form a barrel-like structure; the caspase- (C-L; LLE/β1), trypsin- (T-L; LRR/β2), and chymotrypsin- (CT-L; LLVY/β5) like peptidase activities are located at the β1, β2, and β5 proteasomal subunits, respectively. The 19S RP is involved in substrate recognition, deubiquitination, unfolding and translocation of proteins into the 20S CP [9].

The catalytic activity of the proteasome is central to quality control of protein synthesis as non-functional newly synthesised polypeptides originating from cytosolic or ER-bound ribosomes are targeted for degradation to cytosolic or ER-bound proteasomes [ER associated protein degradation (ERAD)] respectively [32]. Proteasomes are also found in the nucleus, where they participate in DNA damage response (DDR) processes and in the outer membrane of the mitochondria, where they execute outer mitochondrial membrane-associated degradation (OMMAD) during the activation of the UPR^mt^ [33]. UPS is also involved in the degradation of mitochondrial fusion/fission proteins [2] and, thus, apart from genome and proteome stability, UPS functionality is also critical for mitostasis maintenance.

## 4. Stress and Proteome Damage Responses: The Nrf2 Transcription Factor

The various branches of the PN are functionally coordinated by different signalling cascades, which sense and respond to imbalances in proteostasis and/or increased amounts of stressors [8,34]. These signalling cascades are modulated by stress sensitive, short-lived transcription factors [21,35,36].

**Figure 1:**
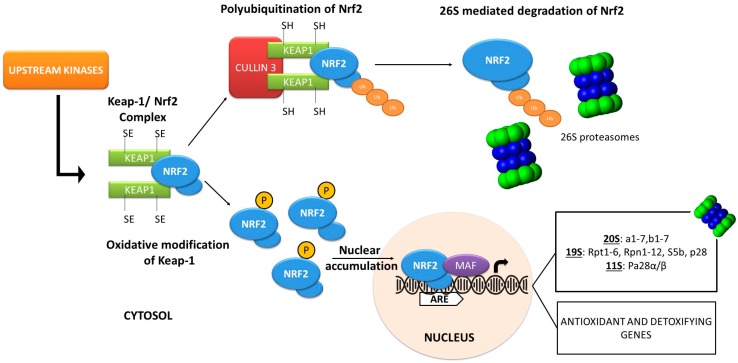
Schematic presentation of the pathways known to modulate Nrf2 activity. Under basal conditions, Nrf2 is poly-ubiquitinated and targeted for proteasomal degradation. Oxidative stress modifies Keap1 leading to Nrf2 stabilization and nuclear translocation. In the nucleus, Nrf2 activates a wide range of transcriptional targets including antioxidant and proteostatic genes. The Nrf2 activity can be also modulated by upstream kinases (e.g., Akt or Gsk-3).

As mentioned, one of the several proteins that comprise the network of stress-responsive cellular sensors is the transcription factor Nrf2, which plays a central role in cellular responses against oxidative and/or xenobiotic damage (Figure 1) [24]. Under physiological conditions, Nrf2 is a short-lived protein, because it is constantly targeted by Kelch-like ECH- associated protein 1 (Keap1) for Ub-dependent proteasomal degradation; parallel to Keap1, the Beta-transducin repeat-containing protein/glycogen synthase kinase-3 (b-TRCP/Gsk-3) axis can also mediate degradation, and thus inhibition, of Nrf2 [37]. In response to increased amounts of oxidants, the three cysteine residues Cys-151, Cys-273 and Cys-288 of Keap1 are oxidised leading to reduced Keap1-Nrf2 binding and consequently to reduced Nrf2 ubiquitination [38]. This event stabilises Nrf2, which then translocates from the cytosol to the nucleus to form a heterodimer with its partner v-Maf avian musculoaponeurotic fibrosarcoma oncogene homolog (Maf); the dimer binds to the antioxidant response elements (AREs) or electrophile response elements- (EpREs) of the DNA and regulates the expression of numerous genes [39,40]. The Nrf2 signalling pathway provides detoxification through the elimination of potentially harmful and toxic compounds or metabolism derivatives [24,41]. Reportedly, in higher metazoans, Nrf2 is also involved in the upregulation of UPS and ALP genes during proteotoxic stress [42,43,44,45].

## 5. The Autophagy-Lysosome Pathway (ALP)

The ALP is, next to the UPS, one of the two main cellular degradation and recycling mechanisms in eukaryotic cells [46]. Macroautophagy (hereafter referred to as autophagy) describes enclosure of cellular material in double-membrane vesicles, also known as autophagosomes, and subsequent fusion with lysosomes for degradation [47]. The process of autophagy can be summarised in five events, namely induction, nucleation, expansion, fusion and cargo degradation/recycling [46].

Cellular energy levels, as well as nutrients and growth factors, are major players in the regulation of autophagy since the primordial function of autophagy is to protect cells from starvation-associated stress [47,48]. mTOR (mammalian target of rapamycin), a serine-threonine kinase, is the main regulator of cellular metabolism and controls anabolic as well as catabolic processes in response to environmental stimuli [48]. Consequently, mTOR activity also influences autophagy regulation [49].

Downregulation of mTOR activity under conditions of nutrient deficiency or growth factor depletion results in the induction of autophagy [50]. mTOR forms two distinct complexes, mTORC1 and mTORC2 [48]. Repressed mTORC1 signalling activates the autophagy-initiating UNC-5 like autophagy activation kinase (ULK) complex, which includes ULK1/2, FIP200, autophagy-related gene 101 (*ATG101*) and the autophagy-related gene 13 (*ATG13*) [47]. ULK1/2 and *ATG13* are targets of mTORC1 phosphorylation that renders them inactive. Upon mTORC1 inhibition, these molecules are not phosphorylated and ULK kinase activity is restored leading to the recruitment of the Vps34 complex to the phagophore, a double membrane intermediate [46,47]. The recruitment of Vps34 initiates the binding of other ATG proteins and the lipidation of LC3 [47]. Upon binding of ATG12 and LC3, the phagophore expands [46]. The autophagosomes are fused with lysosomes, initiating the last step of the autophagy process, in which the cargo is degraded by hydrolases and macromolecules are released back into the cytoplasm [46]. The molecular mechanisms of mTORC2 regulation are less well understood [48].

## 6. Upstream Regulators of Autophagy-Lysosome Pathway

**Figure 2:**
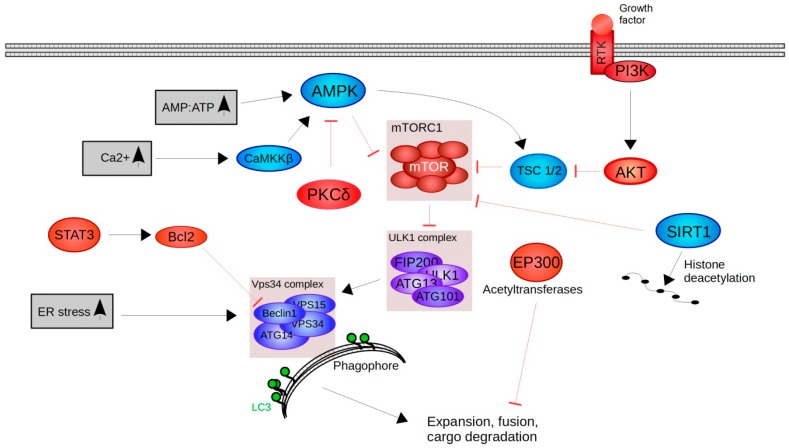
Mechanisms of autophagy-induction. The mTORC1 complex is a central regulator of autophagy-initiation. If active, mTORC1 inhibits the ULK1 complex and its ability to recruit the Vps34 complex to the phagophore. The binding of the Vps34 complex is essential for lipidation of LC3, autophagosome formation, fusion and consequent cargo degradation. Cellular stressors (**shown in grey**), such as ER stress, increasing intracellular Ca^2+^ levels and the shift of energy levels (increase of the AMP:ATP ratio) influences autophagy initiation either upstream of mTORC1 or directly at the Vps34 complex. Other positive regulators of autophagy (**shown in blue**) include SIRT1 and TSC1/2 signalling. Negative regulators of autophagy (**shown in red**) comprise PI3K/Akt, STAT3/Bcl2, PKCδ and EP300.

As mentioned above, mTOR inhibition leads to activation of autophagy. Consequently, regulators of mTORC1 play a major role in autophagy regulation. The growth factor (GF)/phosphoinositide 3-kinase (PI3K)/protein kinase B (Akt) signalling pathway is a well-established upstream regulator of mTORC1 [48]. Activated Akt is able to repress the potent mTORC1 inhibitor TSC1/2, which means that if GF/PI3K/Akt signaling is active, also mTORC1 is active [51] (Figure 2).

Another mTORC1 upstream regulator is AMPK, which is able to sense changes in cellular energy levels and is activated by increased AMP:ATP ratio [46]. Active AMPK phosphorylates and consequently inhibits mTORC1, stimulates TSC1/2 and activates ULK1 by direct phosphorylation [46]. In addition, AMPK is sensitive to intracellular Ca^2+^ levels via the calmodulin-dependent protein kinase kinase-β (CaMKKβ). Increasing cytosolic Ca^2+^ levels activate CaMKKβ, which in turn stimulates AMPK signalling [52].

As mentioned above, the ER is a membrane-bound organelle responsible for post-translational modifications and the correct folding of secretory and/or membrane proteins [53]. Additionally, the ER is a key player in lipid biosynthesis, intracellular Ca^2+^ balance and energy metabolism [53]. If ER homeostasis is disrupted (referred to as ER-stress) unfolded or misfolded proteins begin to accumulate resulting in increased autophagic activity [53,54].

Another probable mechanism for autophagy induction is the signal transducer and activator of the transcription 3 (Stat3)/Bcl-2 pathway [55]. Stat3 upregulates the pro-survival gene Bcl-2, which is known to prevent cells from undergoing autophagy by directly binding to Beclin-1 [56]. Consequently, inhibition of Stat3/Bcl-2 in esophageal squamous-cell carcinoma cells has the opposite effect on Beclin-1 regulation and induces autophagy [55].

Sirtuins are members of a highly conserved family of NAD^+^-dependent (class III) protein deacetylases [57]. SIRT-1 deacetylates histones and non-histone proteins including transcription factors and consequently regulates a broad range of different pathways and cellular processes, such as cell survival, stress resistance and metabolism [58]. Distinct transcription factors affected by SIRT1 signalling are nuclear factor NF-κB, FoxOs and PPAR-gamma co-activator 1α (PGC1α) [57]. This indicates that post-translational modifications, specifically acetylation and deacetylation of proteins, have the potential to regulate autophagy [50]. EP300, for example, acetylates and by this inhibits several autophagy-related proteins [59]. Hence, repressors of EP300 are considered to be autophagy regulating molecules [60].

Furthermore, protein kinase delta (PKCδ) stimulates the expression of tissue transglutaminase 2 (TG2), which is known as a potent repressor of autophagy [61]. The PKCδ/TG2 axis affects other signalling pathways known for autophagy-inhibition, like mTOR, NF-kB and Bcl-2 [61]. Consequently, inhibitors of PKCδ have the potential to activate the autophagy machinery [51].

Evidence suggests that autophagy and ageing are correlated processes since the induction of autophagy seems to extend health- and lifespan, whereas autophagy deficiency accelerates ageing [50,62]. Therefore, the field of research around natural compounds, which are able to promote healthy ageing by manipulating UPS and ALP has gained interest. In the following sections, natural compounds that were found to activate proteostatic modules and to also delay cellular senescence and increase organismal healthspan and/or lifespan will be discussed in detail.

## 7. Natural Compounds Found to Activate Nrf2, UPS or ALP

Natural compounds that affect both the UPS and ALP activity are of particular interest. These bifunctional compounds are summarised in Table 1 and will be discussed in the following section.

Resveratrol(3,5,4′- trihydroxy-trans-stilbene) is a natural polyphenol that occurs in many plants including grapes, berries, knotweed and peanuts [63]. It was first isolated from the roots of *Veratrum grandiflorum* and has numerous beneficial effects on health such as anti-inflammatory, anti-oxidant and cytoprotective properties [58,64,65,66]. Resveratrol promoted longevity in *Saccharomyces cerevisiae, Drosophila melanogaster, Apis mellifera* and *Caenorhabditis elegans,* in the short-lived fish species *Nothobranchius furzeri* and *Nothobranchius guentheri* as well as in metabolically compromised (but not in healthy) mice [57]. This compound is also known for its potential to slow the progression of many age-related human diseases, such as some types of cancer, neurological disorders (e.g., Alzheimer’s disease), cardiovascular diseases and diabetes [58]. Resveratrol induces autophagy through different signalling pathways, depending on the cellular and environmental context [51,65]. In a Parkinson’s disease cellular model, of dopaminergic neuronal cell lines including SH-SY5Y and PC12 cells, 24 h treatment with 50 µM resveratrol promoted AMPK phosphorylation at Thr-172, inhibition of mTOR and also activation of SIRT1 [67]. These mechanisms ultimately contribute to the induction of autophagy [67]. Similar results were achieved in an Alzheimer’s-disease model (APP-HEK293 cells), where a 72 h treatment with 40 µM resveratrol-induced phosphorylation of AMPK at Thr-172 via the Ca^2+^/CaMKKβ-dependent mechanism. Park et al. [68] recently reported that resveratrol inhibited mTOR function by an ATP competitive mode of action in different cancer cell lines. In this study, it was suggested that resveratrol binds directly to the mTOR ATP-binding site and thereby inhibits the complex’s signalling and activates autophagy [68]. Apart from autophagy, two distinct studies on rat models revealed the Nrf2-activating ability of resveratrol. Oral administration of resveratrol in a rat model at a dose of 10 mg/kg of body weight improved antioxidant responses by activating the Nrf2 pathway along with SIRT1 and AMPK [69]. Moreover, treatment of diabetic rats with resveratrol at a dose of 20 mg/kg of body weight revealed that challenging the cells’ redox balance, alternates the phosphorylation status and enhances the transcription of antioxidant enzymes. Changes in the regulation of gene expression were accompanied by the nuclear accumulation of the transcription factors Nrf2 and NF-kB [70].

Metformin, which derives from guanidine, and is found in *Galega officinalis* (Fabaceae) was reported to delay age-related loss of locomotion and lipofuscin accumulation, and to significantly extend median lifespan in *C. elegans* by 40%, when administrated at a concentration of 50 mM. It was shown that the healthspan benefits of metformin could be mediated through the activation of the Skin1/Nrf2 transcription factor [71]. In support, Martin-Montalvo et al. [72] reported that metformin improved healthspan and lifespan in mice via the induction of the Nrf2-ARE pathway. In terms of human disease treatment, metformin is currently used as a first-line drug to treat diabetes type 2 [73]. Interestingly, metformin administration does not only affect diabetes mellitus type 2 but it also improves other clinical conditions, such as cardiovascular disease [74]. It was suggested that autophagy could be the mechanism underlying the reduction of diabetic cardiomyopathy [55,75]. AMPK phosphorylation at Thr-172 and consequent inhibition of mTOR signalling seem to be the key regulators in metformin-induced autophagy in melanoma cells [76]. As shown by Feng et al. [55] in esophageal squamous cell carcinoma, treatment with 10 mM metformin for 48 h activated autophagy in an AMPK-dependent as well as in AMPK-independent manner. In this context, the AMPK-independent signalling pathway involves STAT3 inhibition; repression of Bcl-2 and therefore autophagy activation [55].

Curcumin (diferuloylmethane) is synthesised by *Curcuma longa* (also known as turmeric) and has antioxidant, anti-inflammatory, anticancer, neuro- and cardio-protective properties [77,78]. The treatment of *Caenorhabditis elegans, Drosophila melanogaster* and *Mus musculus* with curcumin proved the compound’s potential to increase lifespan [79]. In these studies, the mean lifespan of *Drosophila* flies was extended by more than 10% via the addition of 0.5–1.0 mg/g of curcumin in the culture medium by suppressing oxidative stress and lipid peroxidation, reducing the accumulation of dialdehydes and improving locomotor performance; these health beneficial effects have been attributed to the modulation of a number of stress-responsive genes including the antioxidant enzyme superoxide dismutase [80,81]. At the molecular level, it was found that curcumin could upregulate the sirtuin pathway and strongly activated the Nrf2-ARE pathway in different tissues [82,83].

Additionally, it was demonstrated that curcumin (or its analogue bis-dehydroxy-curcumin) induced apoptosis via autophagy activation in various types of cancer cells including colon cancer, uterine leiomyosarcoma, ovarian cancer and lung adenocarcinoma [54,77,84,85,86]. Curcumin or its analogues, B19 or bis-dehydroxy-curcumin, were reported to induce autophagy either via the ER-stress mediated pathway, as shown in ovarian cancer cells [54] and colon cancer cells [84] by acting as an inhibitor of EP300 acetyltransferases in multiple cancer cells, or through mTOR inhibition, as shown in uterine leiomyosarcoma cells [87].

Genistein (4′, 5, 7-trihydroxyisoflavone) is a natural polyphenol found in soy and other legumes, such as *Vigna angularis,* that reportedly extended the lifespan of *Caenorhabditis elegans* [88,89]. Skin anti-ageing effects have been accredited to this polyphenol, as it can bind to estrogen receptors, enhance collagen biosynthesis, act as an antioxidant and protect from UV-induced photodamage [88]. Additionally, the enrichment of rat diet with low doses of genistein demonstrated neuroprotective properties along with antioxidant and cognitive function preservation effects [90]. Genistein was found to enhance endothelial nitric oxide synthase (eNOS) activation; the Nrf2/HO-1 pathway via increased Keap1 S-nitrosylation as well as the nuclear accumulation and DNA binding activity of Nrf2 [90]. Genistein was also found to protect cerebrovascular endothelial cells from oxidative damage via the activation of the Nrf2-mediated stress response pathway by acting on upstream kinases such as PI3K [91]. Genistein induced autophagy in MCF-7 cells (100 µM genistein for 72 h), as well as in ovarian cancer cells (100 µM genistein for 24 h) [92,93]. This compound can affect numerous cellular processes, due to its potential to repress the activity of different protein-tyrosine kinases [93]. Consequently, genistein influenced PI3K-Akt signalling, which might contribute to a lower rate of glucose metabolism and hence, to a starvation-like autophagy induction in ovarian cancer cells [51,93].

Catechins are polyphenols synthesised by *Camilla sinensis* [94]. Catechins are known to exert antioxidant, anti-inflammatory and anti-angiogenic properties and have demonstrated beneficial effects in treating and preventing cancer, diabetes, obesity, neurodegenerative disease and cardiovascular diseases [95]. Furthermore, the cosmetic industry shows a growing interest in these polyphenols, since they have demonstrated skin anti-ageing effects [96]. Catechins and their derivatives were able to extend lifespan in *Caenorhabditis elegans, Drosophila melanogaster* and *Mus musculus* [97,98]. More specifically, Sucro-Laos et al. [99] and Sunagawa et al. [100] found that methylated epicatechin derivatives (200 μΜ) prolonged the mean lifespan of *C. elegans* by ~6–12%, probably by modulating an energy-intensive, stress response and repair system [101]. Moreover, daily administration of epigallocatechin gallate (EGCG) (55 and 220 μM) in *C. elegans* prolonged the mean lifespan of the worm by ~10%. It also significantly decreased intracellular oxidative stress and formation of lipofuscin, most likely via the activation of the Daf16/FoxO signalling pathway [102,103]. In addition, both in vitro and in vivo studies found EGCG to induce Nrf2 expression exerting cancer chemopreventive properties [104] and protection against lupus nephritis [105]. EGCG has also demonstrated its protective effect on human umbilical vein endothelial cells from PM2.5-induced oxidative stress by upregulating Nrf2/HO-1 via the activation of the p38 MAPK and the extracellular signal-regulated kinase 1/2 (ERK1/2) signalling pathways [106]. Furthermore, Na et al. [107] found that EGCG induces Nrf2-mediated expression of manganese superoxide dismutase (MnSOD) and HO-1 and activated ERK1/2 and PI3K/Akt signalling in MCF10A cells. Moreover, EGCG was found to promote the dissociation of Nrf2 from Keap1 [108]; in support, Kanzaki et al. [109] demonstrated that sulforaphane and EGCG augmented the nuclear translocation of Nrf2 and HO-1 expression in a mouse monocytic cell line. Also, Yang et al. [106] reported that EGCG increased the Nrf2 nuclear translocation in a normal rat kidney proximal tubular epithelial cell line, namely NRK-52E.

Regarding autophagy, the action of catechins is ambiguous, concentration- and cell type-dependent [110]. For example, 100 µM catechin blocks hypoxia/reperfusion-induced autophagy via increasing Akt/mTOR phosphorylation in microglia [111]. On the other hand, Kim et al. [112] showed that treatment with 10 µM EGCG for 4 h induces autophagy through a Ca^2+^/CaMKKβ/AMPK-mediated mechanism in endothelial cells.

## 8. Plant-Derived Natural Compounds Specifically Modulating Nrf2 and/or Proteasome Activity

The activation of the UPS system by natural compounds, either directly or via its master regulator Nrf2, has been reviewed in several cellular models, e.g., primary cultures of mammalian origin, as well as in yeast, worms, flies, rodents and humans and has been shown to result in extended cellular viability, lifespan extension and/or resistance to various types of stressors [9,114]; compounds with the potential to activate UPS or modulate Nrf2 activity are summarised in Table 2.

Sulforaphane is a natural product within the group of organosulfur compounds (glycosinolates) that can be isolated from cooked cruciferous vegetables and is present in a variety of oral supplements, containing its purified form or in broccoli sprout extract [115,116,117,118]. Sulforaphane has been characterised for its anti-cancer, anti-oxidant and anti-microbial properties most of which are attributed to its ability to activate the Keap1-Nrf2-ARE pathway [119]. The treatment of human HEK293 cells with 5–20 μΜ of sulforaphane enhanced Nrf2 nuclear translocation by directly modifying Keap1 cysteines [38,119,120,121,122]. Acute or long-term administration of sulforaphane induces antioxidant and phase II drug metabolizing enzymes in several tissues [123,124,125,126,127]. Sulforaphane has also been found to exert chemopreventive effects against carcinogenesis in various types of carcinogen-induced and transgenic cancer models [91].

Reserpine, an indole alkaloid, is another compound from the group of glycosinolates, which is isolated from the dried root of *Rauwolfia serpentine* (Apocynaceae). Chronic administration (30 μΜ) of reserpine to *C. elegans* increased its lifespan by 31%, provided stress tolerance and delayed age-related decline in the mobility of the worm [91]. These results are further supported by another study, which suggests that reserpine exerted cancer-preventive properties by reactivating Nrf2 and inducing the expression of cytoprotective genes [128]. Although the use of reserpine as an anti-ageing factor is rather promising, according to Drugs.com (Drugs Information Database) the clinical use of reserpine as anti-psychotic and anti-hypertensive drug (0.1–0.5 mg orally daily) revealed numerous side-effects, such as respiratory, psychiatric and cardiovascular complications.

Andrographolide, isolated from *Andrographis paniculata* (Acanthaceae) is a diterpene lactone compound that exhibits neuroprotective properties in both in vitro and in vivo experimental models of stroke [129]. Part of andrographolide’s neuroprotective mechanism was attributed to the increase of the 2-heme oxygenase (HO-1) (Nrf2 transcriptional target) expression through p38-mitogen-activated protein kinase (MAPK) regulation, after the intraperitoneal injection of middle cerebral artery occlusion (MCAO)-insulted rats with 0.1 mg of andrographolide per kg [130].

Phenolic compounds, are well known dietary supplements for several conditions but they should be carefully used after medical consultation due to side effects resulting from high dosing or potential pharmacological interactions with conventional therapies [131]. Besides the aforementioned curcumin, catechins and genistein other phenolic compounds were described to modulate proteasome activity and/or Nrf2 activity, as follows.

Cinnamic aldehyde (flavonoid) and pterostilbene (stilbenoid) are well-known Nrf2 activators used to treat diabetes [132]. Cinnamic aldehyde acts either directly on the Nrf2–Keap1 complex by modifying critical cysteine thiol residues of Keap1 or on upstream kinases such as Akt, ERK, PI3K, protein kinase C- (PKC) and c-Jun *N*-terminal kinase- (JNK) causing the release of Nrf2 from Keap1. On the other hand, the mechanism of pterostilbene is not well understood; however, it has been proposed that it increases the expression of target genes downstream of Nrf2 by promoting Nrf2 expression and nuclear translocation.

Oleuropein (phenylethanoid), the major constituent of *Olea europea* (Oleaceae) leaf extract, is a well-known natural compound with anti-tumour and anti-inflammatory properties due to its anti-oxidant potency and radical scavenging ability [133]. It was also found to suppress oxidative stress, reduce protein oxidation and increase all three proteasome activities in human embryonic fibroblasts at a concentration of 0.5 μg/mL; moreover, it was found to delay cellular senescence by approximately 15% [134]. Interestingly, in contrast to other proteasome activators with either relatively low potency or high molecular mass, oleuropein is a small molecule that activates the proteasome at low concentrations [135].

Osthole, a compound from the coumarin group isolated from the seeds of *Cnidium monnieri* (Apiaceae) presents a variety of health-beneficial properties such as neuroprotection, hepatoprotection, cardiovascular protection, prevention against cancer, inflammation and infections and modulation of the immune system [136]. In fact the administration of osthole (daily dose of 30 mg/kg body weight by intraperitoneal injection) improved an accelerated, early stage, focal segmental glomerulosclerosis mouse model by activating Nrf2 and subsequently inhibiting nuclear factor kappa-light-chain-enhancer of activated B cells- (NF-κB)-mediated cyclooxygenase 2- (Cox-2) expression and apoptosis [137]. In addition, osthole was shown to reduce inflammation induced by lipopolysaccharide in BV2 cells in a dose-dependent manner via among others the activation of the Nrf2 pathway [138]. Increased protein levels of Nrf2 and HO-1 along with the neuroprotective properties of this compound were revealed after the treatment of C57 BL/6J mice with transient global brain ischemia with 100 mg/kg of osthole [139].

Last but not least among the phenolic acids that have been suggested to act as Nrf2 activators [140]; rosmarinic acid (at 200 μM) extended lifespan (by ~11%) and enhanced thermotolerance of *C. elegans* via its antioxidant properties [141].

Finally it should be mentioned that although the compounds described above have been scientifically proven to act as Nrf2 or proteasome activators in several model organisms (Table 2) they have not been employed in anti-ageing oriented clinical trials yet. According to clinicaltrials.gov coordinated by the U.S. National Library of Medicine (NIH) most of them have been examined as therapies for age-related disorders, such as cancer, diabetes mellitus type 2 and cardiovascular and neurodegenerative diseases but not as factors that could potentially extend healthy ageing or delay the onset of age-related diseases. Several challenges have to be faced in order to extrapolate these studies from model organisms to humans due to the complex human physiology, the diversity of human ageing phenotypes, potential adverse effects and, most important, the need for well-defined, measurable, ageing or age-associated outcomes [142].

## 9. Natural Compounds That Act as Autophagy Inducers

**Figure 3:**
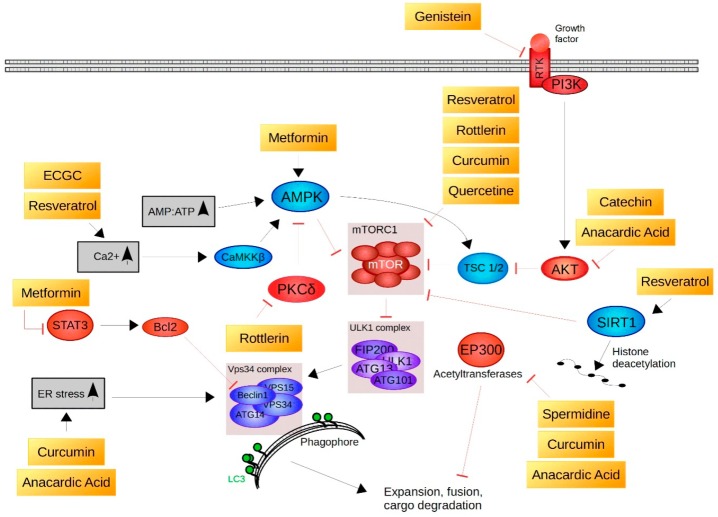
Natural compounds driving autophagy initiation. Plant-derived compounds (**shown in yellow**) induce autophagy by influencing cellular stressors (**shown in grey**); by activating positive regulators (**shown in blue**), or by repressing negative regulators (**shown in red**) of autophagy.

In the following section, we review the main compounds found to activate autophagy (Table 3) and consequently prolong lifespan in various models. The molecular targets (where known) for these compounds in mammalian cells are shown in Figure 3.

Spermidine (*N*-(3-aminopropyl) butane-1,4-diamine) is a natural polyamine that is synthesised by and present in many plants, animals and also humans [144]. Treatment with spermidine extends the life expectancy of *Saccharomyces cerevisiae, Caenorhabditis elegans, Drosophila melanogaster, Mus musculus* as well as the replicative potential of normal human cells and improves cardiac function in rodent models [145,146]. Autophagy activation was reported to be critical for spermidine-mediated increased longevity and for the cardioprotective effects in mice and humans [146,147]. Pietrocola et al. [60] recently suggested that incubation of human osteosarcoma U2OS cells with 100 µM spermidine for 4 h activates the autophagy machinery by inhibiting acetyltransferases such as EP300. EP300 senses nutrient-dependent acetyl-CoA levels and inhibits important autophagy regulators including ATG5, ATG7, ATG12 and LC3 [60]. Regarding neuroprotection, it was suggested that 1 mM spermidine (1 h) prevents staurosporine-induced neuronal injury in PC12 cells (rat pheochromocytoma cells) and rat cortical neurons by inhibiting caspase-3 and therefore blocking the cleavage of Beclin-1, which consequently restores autophagic flux [148].

Quercetin (3,3′,4′,5,7-pentahydroxyflavone) is one of the most common dietary flavonoids found in vegetables and fruits, such as broccoli, onions, cherries, red grapes, apples and berries [149]. It is well known for its antioxidant activity and it was reported to induce apoptosis in numerous cancer cell lines [150]. Additionally, quercetin has been shown to augment resistance to oxidative stress and prolong lifespan in *Saccharomyces cerevisiae* and in *Caenorhabditis elegans* [151,152]. The anti-cellular senescence effect of 2 µg/mL quercetin (55 days of treatment) in human fibroblasts and its potential to rejuvenate senescent cells has been demonstrated [153]. Also, 24 h treatment with 50 µM quercetin induced autophagy by de-phosphorylation and consequent inhibition of mTOR, as well as by downregulating anti-autophagic proteins like c-FLIP and v-FLIP in primary effusion lymphoma and in HeLa cells [154,155].

Rottlerin [1-(6-[(3-acetyl-2,4,6-trihydroxy-5-methylphenyl)methyl]-5,7-dihydroxy-2,2-dimethyl-2H-1-benzopyran-8-yl) −3-phenyl-2-propen-1-one] also known as mallotoxin is a natural polyphenol that can be isolated from *Mallotus philippinensis* [156,157]. Rottlerin might be a potential agent for cancer treatment, e.g., breast cancer [158]. Yet, in spite of the cytotoxic activity of rottlerin against tumour cells, the primary, molecular mechanism of action remains unknown because this compound likely affects many different cellular processes [159]. Interestingly, various studies suggest that this compound is able to induce autophagy in different types of cancer cells [51,160]. Singh et al. [161] reported that 2 µM rottlerin (24 h treatment) activated autophagy in pancreatic cancer stem cells by inhibiting mTOR signalling. In prostate cancer stem cells, it repressed mTOR accompanied by an increase in the expression of autophagy-related proteins including ATG5, ATG7, ATG12 and Beclin-1 [162]. Additionally, it is a selective PKCδ-inhibitor, which in turn leads to NFκB-signalling repression and consequent activation of autophagy in breast, pancreatic and colon cancer cells [51].

Anacardic acid (6-pentadecylsalicylic acid) is synthesised by *Anacardium occidentale* (cashew tree), *Ozora insignis*, *Gingko biloba* and *Amphipterygium adstringens* and is a natural histone acetyltransferase inhibitor, that has antioxidant, anti-inflammatory, antimicrobial and anticancer properties [163,164]. Tan et al. [53] demonstrated that anacardic acid had the potential to induce autophagy in prostate cancer cells via the induction of ER-stress and repression of Akt signalling. Similarly, Seong et al. [165] demonstrated the stimulation of ER-stress related autophagy in lung adenocarcinoma cells A549 upon anacardic acid treatment (3 µg/mL; 24 h). In U2OS cells, neuroglioma cells and also in murine embryonic fibroblasts administration of 50 µM anacardic acid (4 h treatment) induced autophagy by inhibiting EP300 acetyltransferase, which directly represses autophagy proteins, including ATG5, ATG7, ATG12 and LC3 by acetylation [60].

## 10. Concluding Remarks

Considering the rather modest estimation of plants containing ~10^5^ bioactive compounds [166], the number of natural products (NPs) with demonstrated in vivo anti-ageing action remains extremely low. NPs represent an extraordinary inventory of highly diverse structural scaffolds that can be tested as potential chemopreventive agents. Nevertheless, most of the world’s plant, marine, or microbial chemodiversity remains uninvestigated. Thus, carefully designed high-throughput, screening studies to identify compounds with activity against the aforementioned PN targets and downstream testing of selected hits in animal models or in human clinical trials will certainly reveal novel compounds that exert health-promoting properties.

As a note of caution, results obtained with model systems and model organisms cannot necessarily be extrapolated to humans. Another general concern relates to the possibility that some of the compounds described in this review, although showing clear beneficial effects on health-related parameters in model systems or model organisms, may display unexpected adverse effects upon routine application in humans. Whereas negative effects for any of the compounds covered by this review were not explicitly mentioned in the literature, except for quercetin, which causes emesis, nephrotoxicity, hypertension and reduction in serum potassium levels at a concentration of more than 945 mg/m^2^ intravenously injected in cancer patients [149], this does not mean that negative effects may not occur with other compounds as well; in this respect it should be noted that for any kind of compound, excessive doses may bear risks for human health, or, to quote Paracelsus: *Sola dosis facit venenum*. For these reasons, it will be very important in the future to include studies with humans for the most promising natural products.

Preferentially, edible fruits, spices, vegetables or other plant parts (e.g., roots) would be the first choice for these screenings. Furthermore, the identification of non-toxic doses is needed since in most (if not in all) cases, the use of pharmacological PN activators has revealed that the high-level expression of the target is not required for healthspan extension. Rather, mild chronic increases in the activity of proteostatic modules will likely be highly effective for the amelioration of toxicity induced by age-related proteome instability [167]. It is also encouraging that because of the high integrative nature of the PN, small readjustments of a single module (e.g., prevention of the age-dependent decline of proteasome and/or autophagy function) can lead to global beneficial effects indicating the intrinsic propensity of the PN to naturally rebalance as a whole.

In conclusion, tackling ageing and its consequences (increased disability and morbidity) is a much-needed task that, evidently, requires the combined effort of scientists from distinct disciplines including chemistry, pharmacy, biology and medicine. It is foreseen that the progress in understanding the genetic basis of ageing, along with the significant technological improvements in natural products isolation, characterization and tracking of bioactive constituents will lead to identification (e.g., from constituents of the Mediterranean-type diet) of novel natural compounds that will exert healthspan- and/or lifespan-increasing properties and can be thus translated into significant health benefits for humans.

## Figures and Tables

**Table 1:** Bifunctional natural compounds which influence proteasome activity and autophagy.

Compound	Structure	Source	Model	Dose Used	Proposed Mechanism of Action	Reference
Resveratrol	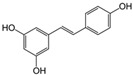	Grape, nuts and peanuts *Veratrum grandiflorum*	Male Wistar Periodontitis rats	10 mg/kg B.W.	↑ Nrf2-mediated antioxidant response	[69]
			Diabetic rats	20 mg/kg B.W.	↑ Nrf2 nuclear translocation	[70]
			SH-SY5Y cells	50 µM—24 h	AMPK	[67]
					SIRT1	
			Various cancer cell lines	50–100 µM—2–4 h	mTOR direct binding	[68]
Metformin	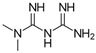	*Galega officinalis*	Male C57BL/6 mice	0.1% *w*/*w*	↑ Nrf2 expression	[72]
			HepG_2_ cells	1.5 mM	↑ Nrf2-mediated antioxidant response	
			*C. elegans*	50 mM	↑ SKN-1 nuclear translocation in the intestine to promote SKN-1-dependent transcription	[71]
			Esophageal squamous cell carcinoma	10 mM—48 h	AMPK	[55]
					STAT3/Bcl-2	
Curcumin	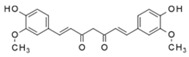	*Curcuma longa*	TRAMP C1 cells	1, 2.5 μΜ	↑ Nrf2 expression	[113]
			*C. elegans*	20 mM	↑ Nrf2-ARE binding	[82,83]
			Uterine leiomyosarcoma cells	50 µM—48 h	Inhibitor of acetyltransferases	[85]
Genistein	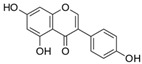	Soy products *Vigna angularis*	Rat model of transient global cerebral ischemia	1 mg/kg B.W.	↑ Modification of Keap1/Nrf2 nuclear translocation	[90]
			Ovarian cancer cells	100 µM—24 h	PI3K-Akt	[93]
			MCF7 cells	100 µM—72 h		[92]
Epigallocatechin gallate (EGCG)	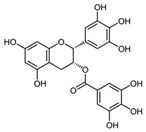	Green tea *Camilla sinensis*	B lymphoblasts	30 μΜ	↑ PI3K/Akt	[113]
			Macrophage foam cells	40 µg/mL	Increased Nrf2-Keap1 dissociation	[108]
			Osteoclast progenitor cells	10 µM	↑ Nrf2 nuclear translocation	[109]
			Human breast epithelial (MCF10A) cells	100 μΜ	↑ Nrf2 nuclear translocation, Nrf2-ARE binding and Nrf2 expression	[107]
			NZB/W F1 lupus-prone mice	120 mg/kg B.W.	↑ Nrf2-mediated antioxidant response	[105]
			Human umbilical vein endothelial cells	50–400 μΜ	↑ Nrf2 and HO-1 expression	[106]
			Endothelial cells	10 µM—4 h	AMPK	[112]

**Table 2:** Natural products modulating Nrf2 and/or ubiquitin-proteasome system (UPS) activity.

Compound	Structure	Source	Model	Dose Used	Proposed Mechanism of Action	Reference
Sulforaphane	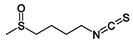	Cruciferous vegetables	Human Keap1-transfected HEK293 cells	5–20 μΜ	Modification of Keap1	[113]
			TRAMP C1 cells	1,2.5 μΜ	↑ Nrf2 expression	
			Mouse embryonic fibroblasts	10 μM	↑ Nrf2 expression	[143]
Reserpine	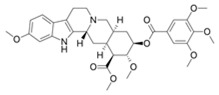	*Rauwolfia serpentine*	Μouse skin epidermal JB6 P + cells	2.5–10 μM	↑ Nrf2 expression	[128]
Andrographolide	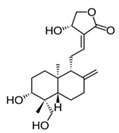	*Andrographis paniculata*	Middle cerebral artery occlusion (MCAO)-insulted rats	0.1 mg/kg B.W.	↑ Nrf2 and HO-1 expression	[129]
Cinnamic aldehyde	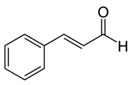	*Cinnamomum verum*	Endothelial cells	100 μM	↑ Nrf2 and HO-1 expression	[113]
			Ηuman epithelial colon cells	10 μΜ	↑ Nrf2-mediated antioxidant response	[132]
			HepG2 cells	100 μM	↑ Nrf2 nuclear translocation	
			STZ-induced diabetic mice (Nrf2^+/+^)	20 mg/kg B.W.	↑ Nrf2 expression	
Pterostilbene	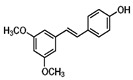	Grapes and blueberries	Male BALB/c mice	5 mg/kg B.W.	↑ Nrf2 and HO-1 expression	[113]
			Tumor xenografts (nude mice) of HEK293T cells	100, 200 mg/kg B.W.	↑ Nrf2 expression	[132]
			INS-1E cells	2–16 μM	↑ Nrf2 activation and expression	
Oleuropein	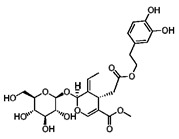	*Olea europa* leaves (green olives, olive leaves, argan oil)	Human embryonic fibroblasts	0.5 μg/mL	↑ In vitro proteasome activities	[134]
					↑ Resistance to oxidative stress and cellular lifespan	
Osthole	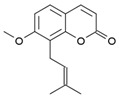	*Cnidium monnerii*	Focal segmental glomerulosclerosis mice	30 mg/kg B.W.	↑ Nrf2-mediated antioxidant response	[137]
			C57 BL/6J mice	100 mg/kg B.W.	↑ Nrf2 protein levels	[137]
Rosmarinic (carnosic) acid	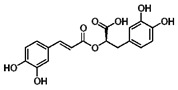	*Rosmarinus officinalis* and *Salvia officinalis*	Mouse model of isoproterenol (ISO)-induced myocardial stress	50, 100 mg/kg B.W.	↑ Nrf2 nuclear translocation	[140]
			*C. elegans*	200 μΜ	↑ Nrf2 mediated antioxidant response	[139]

**Table 3:** Plant extracts and natural compounds activating autophagy.

Compound	Structure	Source	Model	Dose	Proposed Mechanism of Action	Reference
Spermidine	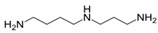	numerous	U2OS, PC12 (rat pheochromocytoma cells)	100 µM—4 h	Inhibitor of acetyltransferases	[60,148]
				1 mM—1 h	Blocks Beclin-1 cleavage	
Quercetin	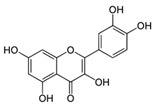	numerous	Primary effusion lymphoma cells	50 µM—24 h	mTOR inhibitor	[154]
					Downregulation of anti-autophagic proteins	
Rottlerin	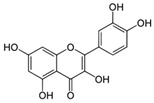	*Mallotus philippinensis*	Pancreatic cancer stem cells	2 µM—24 h	mTOR inhibitor	[162]
					PKCδ inhibitor	
Anacardic acid	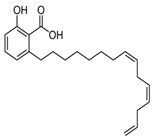	*Anacardium occidentale, Ozora insignis, Gingko biloba, Amphipterygium adstringens*	U2OS, neuroglioma cells, MEF	50 µM—24 h	ER-stress induction	[60]
					Akt	
					Inhibitor of acetyltransferases	
Catechins	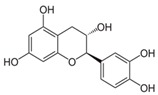	*Camilla sinensis*	Endothelial cells	10 µM—4 h	AMPK	[110]

## Abbreviations

Akt	protein kinase B
ALP	autophagy-lysosome pathway
ARE	antioxidant response element
ATG	autophagy-related gene
b-TRCP	beta-transducin repeat-containing protein
CaMKKβ	calmodulin-dependent protein kinase kinase β
CP	core particle
CR	caloric restriction
EGCG	epigallocatechin gallate
eNOS	endothelial nitric oxide synthase
EpRE	electrophile response element
ER	endoplasmatic reticulum
ERAD	ER associated protein degradation
ERK1/2	extracellular-signal-regulated kinase 1/2
FoxO	forkhead box O
GF	growth factor
Gsk-3	glycogen synthase kinase-3
Hsf1	heat shock factor 1
Keap-1	Kelch-like ECH- associated protein 1
Maf	v-Maf avian musculoaponeurotic fibrosarcoma oncogene homolog
MnSOD	manganese superoxide dismutase
mTOR	mechanistic target of rapamycin
NP	natural product
Nrf2	nuclear factor erythroid 2-related factor 2
OMMAD	outer mitochondrial membrane-associated degradation
OXPHOS	oxidative phosphorylation
PGC-1α	PPAR-gamma co-activator 1α
PI3K	phosphoinositide 3-kinase
PKCδ	protein kinase delta
PN	proteostasis network
ROS	reactive oxygen species
RP	regulatory particle
Stat3	signal transducer and activator of transcription 3
TG2	tissue transglutaminase 2
ULK	UNC-5 like autophagy activation kinase
UPS	ubiquitin-proteasome system
UPRER	unfolded protein response of the ER
UPRmt	unfolded protein response of the mitochondria
